# Tetrahydrocannabinol and Skin Cancer: Analysis of YouTube Videos

**DOI:** 10.2196/26564

**Published:** 2021-05-04

**Authors:** Andrina Mamo, Mindy D Szeto, Roya Mirhossaini, Andrew Fortugno, Robert P Dellavalle

**Affiliations:** 1 Department of Dermatology University of Colorado Anschutz Medical Campus Aurora, CO United States; 2 Department of Medicine Riverside Community Hospital Riverside, CA United States; 3 Department of Medicine University of Tennessee Health Sciences Center Memphis, TN United States

**Keywords:** THC, tetrahydrocannabinol, skin cancer, YouTube, cannabis, social media, internet

## Abstract

**Background:**

Cannabis oil is being used topically by patients with skin cancer as a homeopathic remedy, and has been promoted and popularized on social media, including YouTube. Although topical cannabinoids, especially tetrahydrocannabinol (THC), may have antitumor effects, results from a sparse number of clinical trials and peer-reviewed studies detailing safety and efficacy are still under investigation.

**Objective:**

We sought to assess the accuracy, quality, and reliability of THC oil and skin cancer information available on YouTube.

**Methods:**

The 10 most-viewed videos on THC oil and skin cancer were analyzed with the Global Quality Scale (GQS), DISCERN score, and useful/misleading criteria based on presentation of erroneous and scientifically unproven information. The videos were also inspected for source, length, and audience likes/dislikes. Top comments were additionally examined based on whether they were favorable, unfavorable, or neutral regarding the video content.

**Results:**

All analyzed videos (10/10, 100%) received a GQS score of 1, corresponding to poor quality of content, and 9/10 (90%) videos received a DISCERN score of 0, indicating poor reliability of information presented. All 10 videos were also found to be misleading and not useful according to established criteria. Top comments were largely either favorable (13/27, 48%) or neutral (13/27, 48%) toward the content of the videos, compared to unfavorable (1/27, 4%).

**Conclusions:**

Dermatologists should be aware that the spread of inaccurate information on skin cancer treatment currently exists on popular social media platforms and may lead to detrimental consequences for patients interested in pursuing alternative or homeopathic approaches.

## Introduction

Cannabis oil as a homeopathic remedy for skin cancer was most popularized by Rick Simpson, a Canadian medical marijuana activist. In 2003, Rick Simpson claimed that he was able to cure his basal cell carcinoma with his specially extracted “Rick Simpson Oil,” an illegally produced high-tetrahydrocannabinol (THC) oil made from *Cannabis indica* [1]. An online documentary called Healing Cancer with Cannabis: The Rick Simpson Story [2] currently has over 150,000 views on YouTube and documents Rick Simpson’s journey to curing both his skin cancer and that of others, including basal cell carcinoma and melanoma. Given the increasing importance of social media and YouTube in disseminating information about health care and dermatology [3], we sought to characterize the quality of information patients attain from popular YouTube videos concerning THC and skin cancer.

## Methods

On June 5, 2020, we searched YouTube using the phrase “THC skin cancer.” The ranking option of “view count” was selected. The search resulted in a total of 32 videos; however, only nonduplicate videos with over 1000 views were analyzed in order to obtain accurate representation and capture the most popular videos that had reached the largest YouTube audiences. Two independent reviewers viewed and evaluated all videos, and any discrepancies between reviewers were discussed and resolved in a consensus meeting. All reviewers were experienced in skin cancer pathogenesis, clinical presentation, and treatment. Various predetermined attributes were surveyed, and videos were scored for quality and usefulness with the 5-point Likert scale Global Quality Scale (GQS) as described in Textbox 1 [3,4].

**Global Quality Scale 5-point scale description.**

1. Poor quality, poor flow of the video, most information missing, not at all useful for patients. I would highly discourage a patient with skin cancer from watching this video.
2. Generally poor quality and poor flow. Some information listed but many important topics missing. Of very limited use to patients. I would discourage a patient with skin cancer from watching this video.
3. Moderate quality, suboptimal flow. Some important information is adequately discussed but other information is poorly discussed. Somewhat useful for patients. I would neither encourage nor discourage a patient with skin cancer from watching this video.
4. Good quality and generally good flow. Most of the relevant information is listed, but some topics not covered. Useful for patients. I would encourage a patient with skin cancer to watch this video.
5. Excellent quality and flow, very useful for patients. I would highly encourage a patient with skin cancer to watch this video.

Videos were additionally rated on an adapted DISCERN 5-point reliability scale, an assessment of health information quality used in previous studies [3,5]. Scoring of content accounted for the breadth of skin cancer information discussed, including epidemiology, pathogenesis, clinical features, and treatment. One DISCERN point was earned for each criteria fulfilled, for a maximum of 5 points (Textbox 2).

**Adapted DISCERN content reliability score description.**

**Reliability of information (0 points for no, 1 point for yes)**

1. Are the aims clear and achieved?
2. Are reliable sources of information used? (ie, speaker is a dermatologist, publications were cited)
3. Is the information presented balanced and unbiased?
4. Are additional sources of information listed for patient reference?
5. Are areas of uncertainty mentioned?

Videos were further classified as useful or misleading based on the following yes/no criteria established in prior literature [6-10]:

1. Useful, if they contained scientifically sound information about any aspect of skin cancer.
2. Misleading, if they contained scientifically erroneous or unproven information about any aspect of skin cancer.

For each video, the top 3 comments determined by YouTube according to the number of “thumbs up” ratings were additionally assessed for whether the comment was favorable, neutral, or unfavorable toward the video content. The source and date of the comment were also recorded.

## Results

The 10 videos surveyed (Multimedia Appendix 1) had a total view count of 645,821 views, with an average of 64,582 views per video. Video length ranged from around 2 minutes to over 107 minutes. Sources of videos were varied, and included cannabis companies, Rick Simpson affiliates, and patient perspectives. The surveyed videos had positive social engagement, with a cumulative “thumbs up” score of 4923, and a “thumbs down” score of 183.

Overall, 10/10 videos (100%) had a GQS score of 1, corresponding to “poor quality, poor flow of the video, most information missing, not at all useful for patients.” Just 1 of 10 (10%) videos received a score of 1 of a possible 5 points on the DISCERN scale, corresponding to poor content reliability, while 9/10 (90%) videos received a score of 0. All 10 videos received a “no” rating according to the useful criteria, and a “yes” rating for misleading.

Summaries of video content and top comments for each video are shown in Multimedia Appendix 2. The commenting feature was turned off for one video. Overall, comments were all posted by individual YouTube users, and were largely favorable or neutral toward the video content, with 13/27 comments (48%) classified as favorable, 13/27 (48%) neutral, and 1/27 (4%) unfavorable.

## Discussion

### Principal Findings

Patients are increasingly interested in and selecting nontraditional and alternative therapies for a variety of health conditions. Cannabis oil has achieved great popularity in the past few decades for the treatment of both nonmelanoma and melanoma skin cancers, despite the current lack of evidence; no clinical trials have yet to test their safety and efficacy in humans. Although early preclinical studies have shown that cannabinoids and cannabinoid derivatives may potentially have antitumor effects on keratinocyte carcinoma and melanoma [11], other studies have demonstrated that cannabinoids can be potent proinflammatory chemotactic agents in cell culture models [12].

The body’s endocannabinoid system regulates cell growth through primary endocannabinoids, such as arachidonoyl ethanolamide (AEA) and 2-arachidonoyl glycerol (2-AG), and their metabolism by fatty acid amide hydrolase (FAAH). As detailed in previous reports [13], cannabinoid receptors CB1 and CB2 are found to be expressed on both nonmelanoma and melanoma skin cancers, with the former being largely expressed in the synaptic terminal in order to regulate neurotransmission, and the latter playing a role in activation of psychoactive properties [13].

Melanoma has largely been attributed to chronic sun damage (CSD) as well as non-CSD causes due to mutations in the cell regulatory pathway [14]. A study conducted by Armstrong et al [15] on melanoma cells treated with the cannabinoid THC displayed antitumor properties through the activation of autophagy and apoptotic pathways in vivo and in vitro. Similarly, cannabinoids have displayed antitumor effects in several other studies, focusing on CB2’s anti-inflammatory properties and inhibition of Akt, a key element in the survival pathway of melanomas [16,17].

Cannabinoids have also demonstrated antitumor effects in nonmelanoma skin cancers. Squamous cell carcinomas (SCCs) are some of the most common cancers in humans and have been linked to risk factors including, but not limited to, UV exposure, chemical carcinogens, and viral infections [18]. Induction of apoptosis and tumor regression as an established effect of cannabinoid application has become evident through depression of angiogenic factors, such as vascular endothelial growth factor (VEGF) and angiopoietin-2 (Ang2), as well as decreasing activation of epidermal growth factor receptors (EGF-R) [19].

Although promising research on the treatment of skin cancers with cannabinoids is currently being conducted, the spread of information rooted in evidence-based medicine remains minimal on social media sites such as YouTube. Analysis of video content by two separate reviewers with health care experience and an educational background in skin cancer resulted in assessment of the information presented as uniformly misleading to viewers, along with a GQS of 1 assigned to all videos, demonstrating the pervasiveness of poor-quality information, and also largely unreliable content according to DISCERN criteria. It is concerning that top comments in response to these videos were overwhelmingly either favorable or neutral, highlighting the possibility that fake or secondary individual user accounts could be commenting to generate the outward appearance of validity and support for the video content. With the increasing use of social media, viral content, and thus the immense audience that can be reached via different platforms, including YouTube, inaccurate information can easily be spread to viewers who may be searching for solutions to skin-related problems. It is important for dermatologists to be aware that social media use may subsequently encourage patients to rely on information not provided by trained physicians and health care teams. Potentially harmful side effects or adverse consequences could be experienced by patients due to the dissemination of incorrect or poorly understood information. An increase in the presence of board-certified dermatologists on social media platforms would allow for improved patient education and propagation of medically accurate information to audiences seeking knowledge on skin cancer treatment with cannabinoids.

### Conclusion

This study reiterates the importance of accessible, trustworthy, and engaging educational content curated by medical professionals for patients seeking information about skin cancer treatment online. In the surveyed YouTube data, no videos were curated by medical professionals tackling the popular issue of THC for the treatment of skin cancer, thus highlighting an opportunity for future engagement on social media to improve health education.

## Appendix

Multimedia Appendix 1 Attributes of evaluated YouTube videos.

Multimedia Appendix 2 Content summary and evaluation of the top 3 comments posted for each YouTube video.

## Abbreviations

2-AG: 2-arachidonoyl glycerol
AEA: arachidonoyl ethanolamide
AP-2: angiopoietin-2
CSD: chronic sun damage
EGF-R: epidermal growth factor receptor
FAAH: fatty acid amide hydrolase
GQS: Global Quality Scale
SCC: squamous cell carcinoma
THC: tetrahydrocannabinol
VEGF: vascular endothelial growth factor
