# Supplementary material for: Tetrahydrocannabinol and Skin Cancer: Analysis of YouTube Videos
Source: JMIR Dermatol. 2021 May 4;4(1):e26564. doi: 10.2196/26564 (PMC10501512; doi:10.2196/26564)
Supplement: Multimedia Appendix 1 [file derma_v4i1e26564_app1.docx]

**Multimedia Appendix 1.** Attributes of evaluated YouTube videos.

| Date evaluated | YouTube URL | Video title | Video uploader and identification | Date uploaded | Length (hour, minute, second) | Views | Thumbs up | Thumbs down | GQS score | DISCERN score | Useful? | Misleading? |
| --- | --- | --- | --- | --- | --- | --- | --- | --- | --- | --- | --- | --- |
| 6/5/20 | https://www.youtube.com/watch?v=zD2U4eDBu8A | The Rick Simpson Story: Healing Cancer with Cannabis (more at cureyourowncancer.org) | Uploader: Lincoln Horsley Films  Identification: Individual | 10/29/13 | 0:32:23 | 554,730 | 3977 | 138 | 1 | 1 | No | Yes |
| 6/5/20 | https://www.youtube.com/watch?v=mZRnW5Io_ys | The Cutting-Edge Coconut Oil & Cannabis Cancer Miracle | Uploader: BetterLife  Identification: Individual | 9/13/15 | 0:04.33 | 49,508 | 380 | 35 | 1 | 0 | No | Yes |
| 6/5/20 | https://www.youtube.com/watch?v=theltA8r9eI | Cannabis oil cures skin cancer complete, start to finish | Uploader: Thebestforthecure  Identification: Individual | 7/22/17 | 0:06:01 | 10,435 | 143 | 2 | 1 | 0 | No | Yes |
| 6/5/20 | https://www.youtube.com/watch?v=GiRmciqjiiE | David Triplett Cures His Skin Cancer With Cannabis Oil | Uploader: Cannabis Saves Lives!  Identification: Non-profit channel | 2/1/17 | 0:09:14 | 7687 | 117 | 3 | 1 | 0 | No | Yes |
| 6/5/20 | https://www.youtube.com/watch?v=tkbBU_NjuSQ | Cannabis Oil Cured My Skin Cancer | Uploader: Rick Simpson Phoenix Tears  Identification: individual | 11/29/14 | 0:02:42 | 6371 | 0 | 0 | 1 | 0 | No | Yes |
| 6/5/20 | https://www.youtube.com/watch?v=0coyRnMj7D0 | HOW TO CURE !!!!! SKIN CANCERS !!! - CANNABIS OIL | Uploader: Robbo Da Yobbo  Identification: Individual | 11/1/13 | 0:05:03 | 5066 | 51 | 2 | 1 | 0 | No | Yes |
| 6/5/20 | https://www.youtube.com/watch?v=TfKSB8MARpg | Cannabis Essential Oil Cured my Basal Cell Carcinoma | Uploader: Chelsea Groleau  Identification: Individual | 4/18/18 | 0:05:59 | 3367 | 87 | 0 | 1 | 0 | No | Yes |
| 6/9/20 | https://www.youtube.com/watch?v=3vBjRY6S1GE | Cannabis Oil suppositories and cancer treatment | Uploader: The Goal  Identification: individual | 5/23/19 | 0:06:10 | 2919 | 71 | 0 | 1 | 0 | No | Yes |
| 6/5/20 | https://www.youtube.com/watch?v=mA2Bpz838rw | Skin Cancer and Cannabis: Jack Quinn of Colorado 2014 | Uploader: CannabisPatientNet  Identification: Partnership | 3/26/14 | 0:14:23 | 2899 | 32 | 1 | 1 | 0 | No | Yes |
| 6/16/20 | https://www.youtube.com/watch?v=_mv_7cio3Yk | Corrie Yelland - Reversing Skin Cancer & Anal Cancer Using Cannabis | Uploader: extremehealthradio  Identification: Organization | 3/24/17 | 1:47:51 | 2839 | 65 | 2 | 1 | 0 | No | Yes |
| N/A | Total | N/A | N/A | N/A | N/A | 645,821 | 4923 | 183 |  |  |  |  |
| N/A | Mean (SD) | N/A | N/A | N/A | N/A | 64,582 (172,800) | 492 (1229) | 18 (43) |  |  |  |  |
