# Supplementary material for: Tetrahydrocannabinol and Skin Cancer: Analysis of YouTube Videos
Source: JMIR Dermatol. 2021 May 4;4(1):e26564. doi: 10.2196/26564 (PMC10501512; doi:10.2196/26564)
Supplement: Multimedia Appendix 2 [file derma_v4i1e26564_app2.docx]

**Multimedia Appendix 2.** Content summary and evaluation of the top 3 comments posted for each YouTube video.

| Date evaluated | YouTube URL | Summary of content | Evaluation of Top 3* YouTube Comments (favorable/neutral/unfavorable toward video content) | Date of comment | User commenting | Comment “thumbs up” likes | Comment “thumbs down” dislikes |
| --- | --- | --- | --- | --- | --- | --- | --- |
| 2/16/21 | https://www.youtube.com/watch?v=zD2U4eDBu8A | Individual named Rick Simpson curing his skin cancer with the use of cannabis oil. | Comments for this video were turned off | N/A | N/A | N/A | N/A |
| 2/16/21 | https://www.youtube.com/watch?v=mZRnW5Io_ys | Health benefits of coconut oil and its combination with cannabis to treat cancer. | 1. Neutral | 5 years ago | Individual | 8 | 0 |
|  |  |  | 2. Neutral | 4 years ago | Individual | 6 | 0 |
|  |  |  | 3. Favorable | 4 years ago | Individual | 0 | 0 |
| 2/16/21 | https://www.youtube.com/watch?v=theltA8r9eI | Treatment of squamous cell carcinoma with topical cannabis oil. | 1. Favorable | 2 years ago | Individual | 16 | 0 |
|  |  |  | 2. Neutral | 2 years ago | Individual | 13 | 0 |
|  |  |  | 3. Favorable | 1 year ago | Individual | 7 | 0 |
| 2/16/21 | https://www.youtube.com/watch?v=GiRmciqjiiE | Documentary about the treatment of skin cancer with topical cannabis oil. | 1. Favorable | 2 years ago | Individual | 5 | 0 |
|  |  |  | 2. Favorable | 2 years ago | Individual | 3 | 0 |
|  |  |  | 3. Unfavorable | 1 year ago | Individual | 1 | 0 |
| 2/16/21 | https://www.youtube.com/watch?v=tkbBU_NjuSQ | Medical marijuana used for treatment of skin cancer. | 1. Favorable | 2 years ago | Individual | 5 | 0 |
|  |  |  | 2. Neutral | 2 years ago | Individual | 0 | 0 |
|  |  |  | 3. Neutral | 1 year ago | Individual | 0 | 0 |
| 2/16/21 | https://www.youtube.com/watch?v=0coyRnMj7D0 | Tutorial on how to treat skin cancer with cannabis oil. | 1. Neutral | 1 year ago | Individual | 0 | 0 |
|  |  |  | 2. Favorable | 7 years ago | Individual | 0 | 0 |
|  |  |  | 3. Neutral | 1 year ago | Individual | 0 | 0 |
| 2/16/21 | https://www.youtube.com/watch?v=TfKSB8MARpg | Treatment of basal cell carcinoma with cannabis essential oil. | 1. Neutral | 1 year ago | Individual | 1 | 0 |
|  |  |  | 2. Favorable | 2 years ago | Individual | 0 | 0 |
|  |  |  | 3. Favorable | 1 year ago | Individual | 1 | 0 |
| 2/16/21 | https://www.youtube.com/watch?v=3vBjRY6S1GE | How to use cannabis oil for cancer treatment. | 1. Neutral | 1 year ago | Individual | 2 | 0 |
|  |  |  | 2. Favorable | 5 months ago | Individual | 0 | 0 |
|  |  |  | 3. Neutral | 1 year ago | Individual | 1 | 0 |
| 2/16/21 | https://www.youtube.com/watch?v=mA2Bpz838rw | Treatment of basal cell carcinoma with cannabis oil | 1. Favorable | 6 years ago | Individual | 1 | 0 |
|  |  |  | 2. Favorable | 6 years ago | Individual | 0 | 0 |
|  |  |  | 3. Neutral | 6 years ago | Individual | 0 | 0 |
| 2/16/21 | https://www.youtube.com/watch?v=_mv_7cio3Yk | Treatment of skin & anal cancer with cannabis oil | 1. Favorable | 3 years ago | Individual | 2 | 0 |
|  |  |  | 2. Neutral | 2 months ago | Individual | 0 | 0 |
|  |  |  | 3. Neutral | 3 years ago | Individual | 0 | 0 |

*Top comments were ranked by YouTube according to the number of “thumbs up” ratings, with 1=highest number of thumbs up ratings or likes, 2=second highest, 3=third highest.
